# Quality of place as the winner of the third wave of the COVID-19 pandemic in terms of quality of life. Will this knowledge strengthen the development of geographical psychology?

**DOI:** 10.1016/j.heliyon.2024.e26261

**Published:** 2024-02-10

**Authors:** František Murgaš, František Petrovič, Anna Tirpáková

**Affiliations:** a Department of Geography, Technical University in Liberec, Studentská 2, 461 17, Liberec, Czech Republic; b Department of Ecology and Environmental Sciences, Constantine the Philosopher University in Nitra, Tr. A. Hlinku 1, 949 01, Nitra, Slovakia; c Department of Mathematics, Constantine the Philosopher University in Nitra, Tr. A. Hlinku 1, 949 01, Nitra, Slovakia; d Department of School Education, Faculty of Humanities, Tomas Bata University in Zlín, Štefánikova 5670, Zlín, 760 00, Czech Republic

**Keywords:** COVID-19 pandemic, First wave of pandemic, Third wave of pandemic, Quality of life, Quality of place, Geographical psychology

## Abstract

The paper is focused on the third wave of the pandemic and its comparison with the first wave in terms of the quality of life of university students in Czechia. In the first wave, the society came together, with solidarity being a prominent manifestation. The third wave differed from the first one in medical (vaccination was already available) as well as social terms. The paper has two objectives, the first is to measure the quality of life and related variables in the third wave of the pandemic and to compare the values found in the third wave with those in the first wave of the pandemic. The second objective is to identify which of the factors related to quality of life are predictors of that quality.

The hypothesis assumes different measured values of quality of life for men and women. The measurement yielded knowledge of the high value of quality of place and environmental quality, which can enrich the quality of life epistemology on the one hand and geographical psychology on the other. Trust, health, happiness, quality of place, and safety were identified as factors. The measurement revealed the finding of different quality of life values in the third and first wave of the pandemic. While quality of life values increased in the first wave compared to the pre-pandemic period, both quality of life values and factors decreased in the third wave compared to the first wave. The only exception was one factor that we consider to be a winner of the third wave of the pandemic. The factor that declined in all measurements is considered to be the loser of the third wave of the pandemic. The paper concludes with implications derived from these findings.

## Highlights

• Scientists from many fields have studied the devastating effects of the COVID-19 pandemic on all spheres of human activity. Is there a sphere that has benefited from the pandemic? Yes, it is the concept of quality of place as an objective dimension of quality of life
• The investigation of the quality of life of university students in the third wave of the pandemic showed that on a scale of 0–10 the quality of the place reached an average value of 7.91

## 1 Introduction

In the spring months of 2020, a pandemic of the infectious respiratory disease COVID-19 (hereafter referred to as the pandemic), caused by the SARS-CoV-2 virus, broke out. It was the first global event to affect all countries. The Spanish influenza epidemic of 1918–1920 resulted in a higher number of infections and deaths than the current pandemic as of January 2022, but did not affect all countries. The Second World War or the financial crisis of 2008–2009 affected dozens of countries but not all. At the beginning of the 21st century, viral diseases spread and appeared on several continents - Ebola, Dengue, Zika affected countries in the tropics and subtropics. The respiratory disease SARS affected Canada, Spain, Russia, Brazil, India and Australia, while the respiratory disease MERS affected North African countries, Turkey, Iran and Saudi Arabia a few years after SARS. Both SARS and MERS had effects on countries in Western Europe, the USA and China [[1], [2], [3], [4], [5], [6]]. There are several reasons for the greater spread of these diseases compared to the Spanish flu, one of which can be considered the phenomenon of mass air travel, which has fully manifested in the most recent pandemic.

The effects of pandemic on quality of life and related concepts of well-being or happiness have been addressed by scholars [[7], [8], [9], [10], [11], [12], [13]] and international organizations [[14], [15]]. Naturally, the pandemic has received much attention from authors focused on health-related quality of life [16,17]. Given the evolution of the pandemic over time, the published papers refer to the first wave of the pandemic.

In the paper we focused on the quality of life of university students in Czechia at the time when the third[14] wave of the pandemic was ongoing. A very dynamic development on the one hand and society's reaction to it on the other hand allow us to compare it with the first wave. The pandemic in the individual countries brought scientists from medical disciplines such as virology, microbiology or infectology to the forefront of public interest. They became members of various government pandemic committees. During the pandemic, other disciplines - psychiatry, economics, sociology, geography, mathematics - also came to the fore.

Geographical research of quality of life, well-being, happiness and quality of place [[18], [19], [20], [21], [22]] has led to the emergence of a 'geography of quality of life' (happiness, well-being, etc.). This process is related to the specialisation of geographical research and the related emergence of a number of sub-disciplines [23].

The current boom of interest in quality of life is caused, among other things, by the emergence and rise of positive psychology. Geographers respond to this development by conceptualising the quality of the place as an objective dimension of quality of life. The Concept of 'Geographical Psychology' by psychologist Peter J. Rentfrow speaks in favour of accepting a geographical approach to quality of life. Its essence is the study of the geographical differentiation of psychological phenomena [24].

We seek to investigate the quality of life and related variables in the third wave of the pandemic in a specific group of university students. Interest in quality of life, well-being and happiness is experiencing a boom, part of which is an expanding interest in age groups of population including children [25,26], adolescents [27] and university students [[28], [29], [30]].

In our paper, ‘place’ is considered to be a city or village where students live permanently, not the city where they study. We are interested in which of these variables in the third wave of the pandemic were predictors of quality of life. We consider as predictors those variables for which a change in value affects a change in the value of quality of life.

We stated the following research hypothesis. H1: Both groups of university students, specified by gender, perceive their quality of life differently during the third wave of the pandemic. The paper has two objectives (i) to measure quality of life and its related variables in the third wave of the pandemic and to compare the values found in the third wave with those found in the first wave of the pandemic, (ii) to find out which of the variables related to quality of life are predictors of it.

## 2 Theoretical background

### 2.1 Quality of life

Our approach to quality of life is geographical, we do not deal with how quality of life, well-being or happiness arises, or what enhance or erodes it. We focus on where (in spatial units - district, city, region, country) the quality of life has a low, medium or high value. We obtain this value with a subjective indicator.

In Table 1 we present country ranking in terms of quality of life measurement and the location of the Czech Republic. The table shows the Czech Republic in a very good position of, in fact the best of the countries in Central and Eastern Europe.

**Table 1:** Country ranking in quality of life in selected measurements.

Rank	CEO World (2021)	CEO Happiness	Numbeo (2023)	Legatum (2023)	World Happ. Report (2023)
1.	Finland	Switzerland	Luxembourg	Denmark	Finland
2.	Denmark	Finland	Netherland	Sweden	Denmark
3.	Norway	Iceland	Iceland	Norway	Iceland
4.	Belgium	Netherland	Denmark	Finland	Israel
5.	Sweden	Canada	Finland	Switzerland	Netherland
6.	Switzerland	Norway	Switzerland	Netherland	Sweden
7.	Netherland	Denmark	Oman	Luxembourg	Norway
8.	France	Ireland	Austria	Iceland	Switzerland
9.	Germany	Germany	Norway	Germany	Luxembourg
10.	Japan	Belgium	Spain	New Zealand	New Zealand
Czechia	22.	19.	23.	25.	18.

The quality of life in Czechia stagnated between 2003 and 2015 [36], but began to improve in 2016–2019 [10]. For 2018 Eurostat reported a quality of life in Czechia of 7.4 on a scale of 0–10 [37]. In 2019, i.e. before the onset of the pandemic, it reached 7.38 on a scale of 0–10. Table 2 shows the values of quality of life and variables in 2019 and 2020 in Czechia from various authors.

**Table 2:** Values of quality of life and variables in 2019 and 2020 in Czechia.

Authors	Data of the year	Trust	Health	Quality of life	Happiness	Quality of place	Safety
Petrovič, Murgaš	2019	6.0	8.6	7.4	7.0	7.0	8.0
Murgaš, Petrovič	2020	5.9	8.2	7.6	7.5	7.7	8.1
Petrovič et al.	2020	5.8	8.2	7.6	7.5	7.8	8.1

In 2020, despite expectations of significantly negative pandemic impacts, Petrovič et al. [12] report an average quality of life value of 7.65 on a scale of 0–10 in the first wave of the pandemic in April and May 2020. This is a marginally lower value than the average value of 7.74 measured in 2019. Similar values were found by the Public Opinion Research Centre [38], which measured an average life satisfaction value of 7.28 during 2020 (7.33 in May 2020, 7.38 in June 2020, and 7.13 in December 2020). In the World Happiness Report [39], on a scale of 0–10, Czechia ranked 16th in 2020 with a value of 6.897; the average for the period 2017–2019 is 17th rank and a value of 6.911. The growth in quality of life in the first wave of the pandemic was not specific for Czechia. Some countries (Finland, Germany, USA, Slovakia and Saudi Arabia) achieved higher happiness values in the first year of the pandemic than before it, Croatia even reached 6.508 in 2020 compared to 5.505 as the average for 2017–2019 [40].

### 2.2 Pandemic

The term “pandemic” (from the Greek “pan” for whole, everything and “demos” for people) refers to an epidemic of an infectious disease spread over several continents. The term was first used in connection with the Spanish influenza. The largest number of deaths occurred during the plague epidemic of 1346–1353, also called the “black death”. In Europe, about one-third of the population died in that period. The number of deaths from the four waves of Spanish influenza is estimated at 50 million people worldwide [41], 46,000–88,000 in Czechia [42]. According to the Johns Hopkins University Coronavirus Resource Centre [43], as of October 3, 2023, a total of 6,881,955 people had succumbed to the pandemic worldwide. Some major diseases (circulatory diseases, cancers) with a death toll much higher than the COVID-19 pandemic are not considered pandemics because these diseases are not infectious.

Quality of life or related concepts in the first wave of the pandemic have been investigated by researchers in several countries: in Luxembourg, Germany and Brazil by Abreu et al. [8], in China by Chen et al. [9], in Spain by Iglesias-López et al. [11], in the US state of New Jersey by Murray [44], in Portugal by Gaspar et al. [45], in Czechia the quality of life investigated by Murgaš and Petrovič [10], happiness by Petrovič et al. [12], mental health by Kučera et al. [46], life satisfaction by Public Opinion Research Centre [38] and well-being by Maturkanič et al. [47].

### 2.3 Third wave of the pandemic in Czechia

The third wave of the pandemic broke out in Czechia in the autumn of 2021, according to medical authorities. It differed from the first wave in that vaccination was already available, but was received by the public controversially. From a medical point of view, the third wave differed from the first wave in terms of higher infectiousness and lower mortality [48]. The situation in society in the third wave changed significantly compared to the first wave. The coming together of society in the first wave describe by Murgaš and Petrovič [10] was replaced by its opposite - a division of society into those who accept vaccination and those who reject it. There was aggression, both verbal on social networks and physical, against doctors and media personalities supporting vaccination. At the same time, governments have announced restrictions, bans and fines against the unvaccinated. Mass demonstrations broke out in Czechia as well as in other countries by opponents of vaccination, who considered enforced vaccination an attack on freedom. Peaceful demonstrations often resulted in violent clashes with the police.

## 3 Methodology

The paper is based on the understanding of quality of life as a complex of two dimensions and is focused on the spatial pattern of the quality of life of university students during the third wave of pandemic.

The methodological procedure follows from the set goals and the formulated hypothesis. At the beginning, the measurement of quality of life in the year before the outbreak of the pandemic, in the first and third wave of the pandemic, is outlined. In the paper, we measured the quality of life and other variables of Bachelor's, Master's and Doctoral students in the form of a questionnaire using social networks. Answers were divided into groups of men and women. In quantification, the mathematical statistics methods were used. Basic descriptive statistics for each variable were calculated first. We used parametric tests (*t*-test), which can be used if the assumption of a normal distribution of the sample is met. The Shapiro-Wilk test in Statistica program was used to test the hypothesis of a normal sample distribution.

Subsequently, we constructed histograms of the answers to the question about each variable. The validity of research hypothesis H1 using the nonparametric statistical method "Wilcoxon two-sample test" was verified based on the results obtained using the Shapiro-Wilk test. The average values of the responses to the individual variables were expressed in the form of figures. Using the Statistica program, we calculated the values of the Spearman coefficient of rank correlation between the values of answers to individual questions for both men and women. The implications for the epistemology of quality of life, geography and public policy were drawn from the acquired knowledge.

### Ethical approval

Ethical approval was obtained from the Ethic Committee of the Constantine the Philosopher University in Nitra (chairman Prof. dr. M. Bauerová). The Ethics Committee stated and confirmed that the research does not contradict any ethical rules and confirms that all respondents were informed about the use of their answers. The questionnaire was filled out anonymously. All respondents were initially informed about the objectives of the research and the use of the questionnaire. By filling out the questionnaire, they agreed to its evaluation.

### 3.1 Measurement

One way of obtaining subjective data on quality of life is an online self-report questionnaire [8]. Given the need to limit face-to-face contact in the context of a pandemic, its increased use is to be expected.

In this paper, we investigate quality of life and related phenomena - trust, health, happiness, quality of place and quality of environment of university students in Czechia. Data (N = 203) were collected in the months of October–December 2021, when the third wave of the pandemic was underway, with the collection done using social networks. To avoid refusal to provide data by students, we did not use a face-to-face data collection method.

**Fig. 1:**
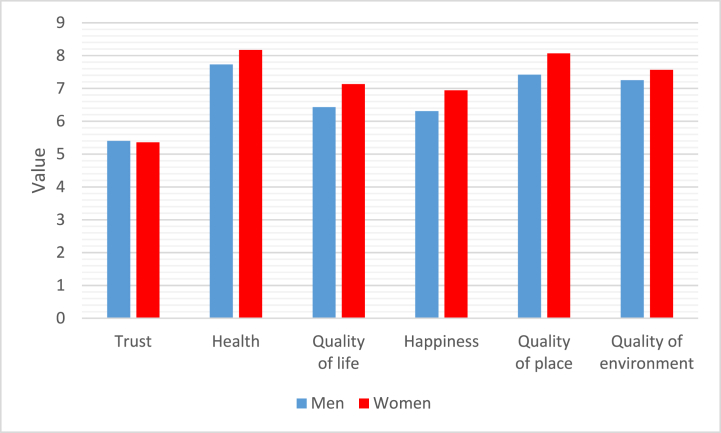
Respondents' answers (averages).

In the first measurement step, we calculated basic descriptive statistics for each indicator, namely the arithmetic mean (Means) and standard deviation (SD) (Table 3) for both groups of respondents (female, male). We also illustrated the results graphically (Fig. 1).

**Table 3:** Descriptive statistics.

	Men		Women		Mean	
	means	SD	means	SD	means	SD
Trust	5.40	±2.04	5.36	±1.83	5.38	±1.88
Health	7.73	±2.38	8.17	±1.76	7.95	±1.94
Quality of life	6.43	±2.62	7.13	±1.99	6.78	±2.19
Happiness	6.31	±2.25	6.94	±2.10	6,78	±2.15
Quality of place	7.42	±2.21	8.07	±1.84	7.91	±1.96
Quality of environment	7.25	±1.56	7.57	±1.83	7.49	±1.76

In Table 3, we can see that the mean values of the answers in the individual questions (indicators) in the two groups of respondents are different. Our aim was to see if these differences between the answers of men and women are also statistically significant. In other words, whether the groups of respondents (men and women) attach different importance to the individual indicators related to the quality of life, whether the above differences in the answers are also statistically significant. In case we would like to use parametric tests (*t*-test) to verify the statistical significance of the differences between the two groups (by gender) in the answers to each question, it is necessary to verify the conditions for the use of parametric methods. A parametric *t*-test can only be used if the assumption of a normal distribution of the sample is met. This assumption can be tested. In our case, we used the Shapiro-Wilk test [49] to test the hypothesis of a normal distribution of the sample set. To test the normality of the distribution of the respective sample set of responses to each question, we will use the Shapiro-Wilk test to test the null hypothesis $H_{0}$: random selection comes from a normal distribution against the alternative hypothesis $H_{1}$: random selection does not come from a normal distribution.

**Fig. 2:**
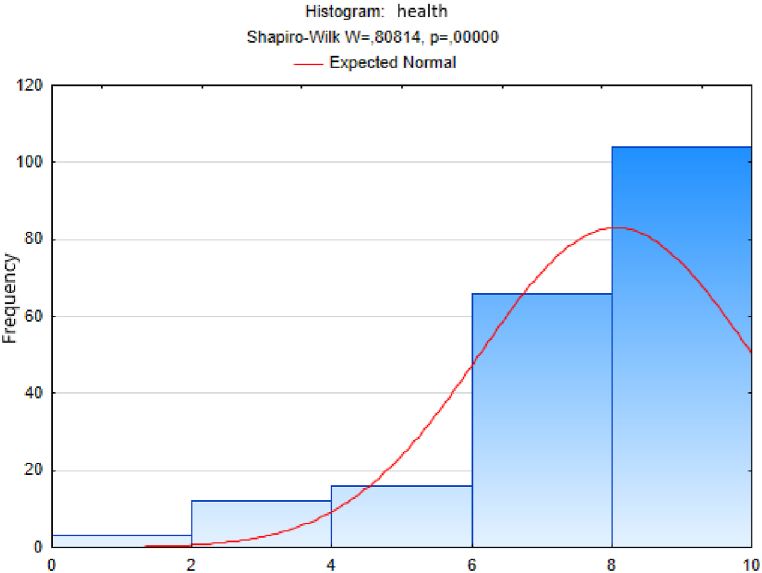
Histogram of answers to question on health.

We first used the Shapiro-Wilk test to test the normality of the distribution of the sample of responses to the question “*On a scale of 0-10, please indicate how healthy/unhealthy you feel*”, using the STATISTICA program. After data entry, we calculated the Shapiro-Wilk test statistic *W* = 0.808 and the probability value *p* = 0.000. We evaluated the test results using the *(p)-*value.^1^ Since the probability *p*-value in our case is less than 0.01, we reject the tested hypothesis $H_{0}$ of a normal distribution of the values of the responses to the health question at the significance level α=0,01. This means that the distribution of values of responses to the quality of life question cannot be considered normal (Fig. 2).

**Fig. 3:**
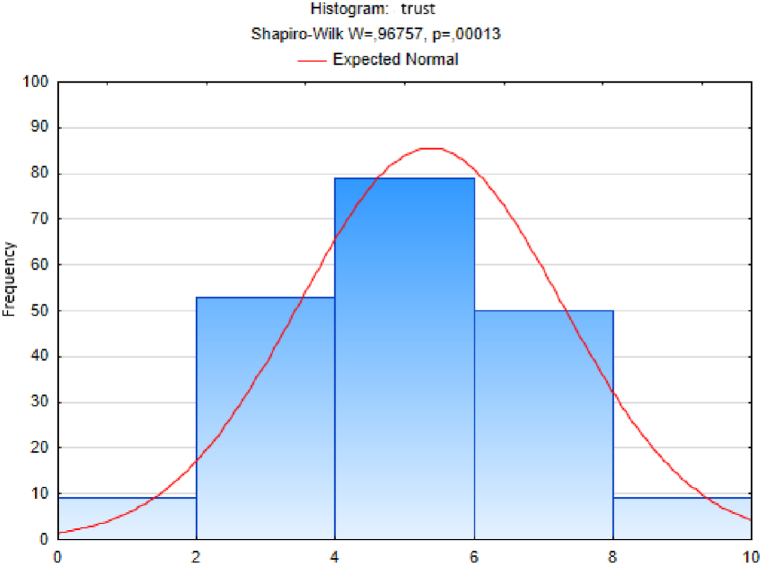
Histogram of answers to question on trust.

**Fig. 4:**
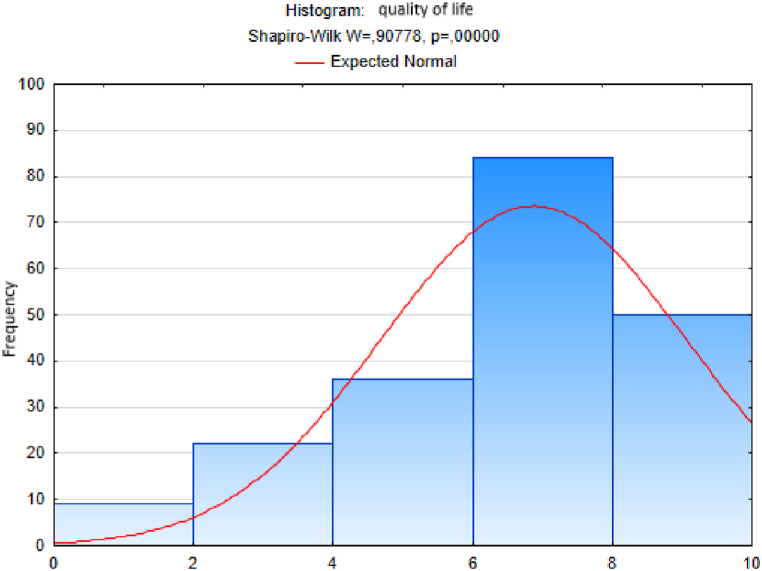
Histogram of answers to question onquality of life.

**Fig. 5:**
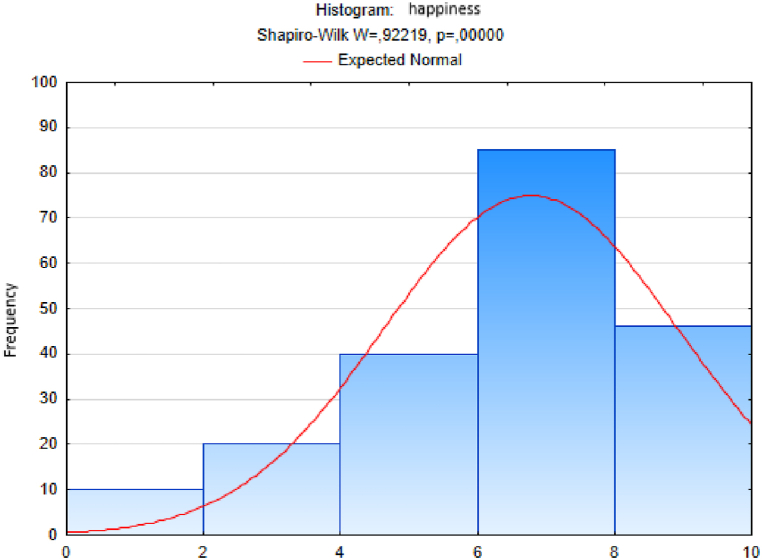
Histogram of answers to question on happiness.

**Fig. 6:**
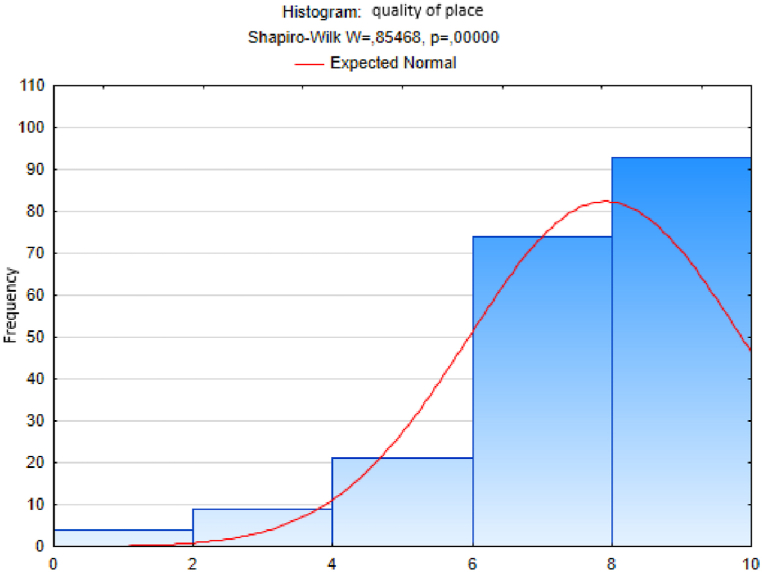
Histogram of answers to question on quality of place.

**Fig. 7:**
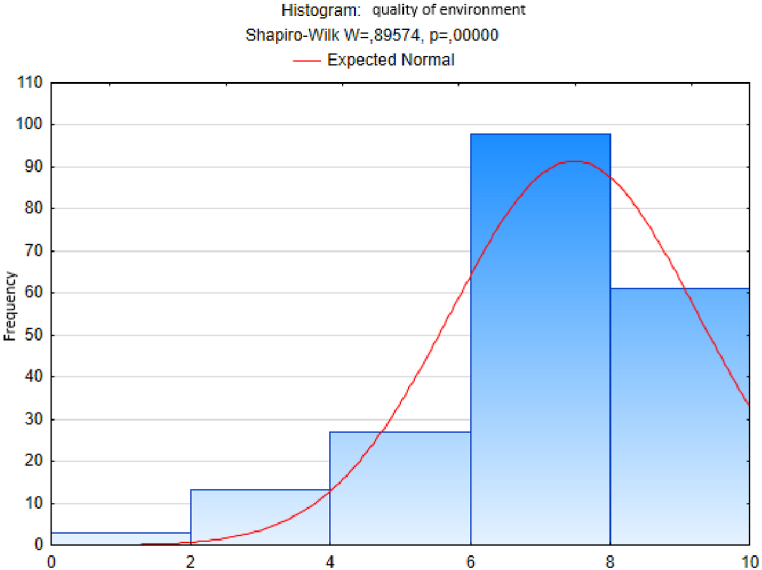
Histogram of answers to question on environment quality.

We followed an analogous procedure to test the hypothesis of a normal distribution of the sample sets of responses to the other questions - on the variables trust, quality of life, happiness, quality of place and quality of environment (Fig. 3, Fig. 4, Fig. 5, Fig. 6, Fig. 7). In these cases too, to test the hypothesis of normal distribution of the respective sample set, we used the Shapiro-Wilk test, which we performed in the STATISTICA program. After entering the data we calculated the value of the Shapiro-Wilk test statistic *W* and the probability value *p* in all cases. The results are shown in Table 4.

**Table 4:** Results of Shapiro -Wilk test.

Variables	W	p
trust	0.967	0.000*
quality of life	0.908	0.000*
happiness	0.922	0.000*
quality of place	0.855	0.000*
quality of environment	0.896	0.000*

Note: * statistically significant value.

Since the probability *p*-value in all cases is less than 0.01, we reject the tested hypothesis $H_{0}$ of a normal distribution of response values for each question, given in Table 4, at the significance level α=0,01. Based on the results of the Shapiro-Wilk test (the results are in Table 4 and graphically shown in Fig. 2, Fig. 3, Fig. 4, Fig. 5, Fig. 6, Fig. 7), none of the observed variable (trust, health, quality of life, happiness, quality of place, quality of environment) hasn't a normal distribution. This means that we cannot consider the distributions of observed variables to be normal.

Based on the results obtained by the Shapiro-Wilk test, we used a non-parametric statistical method, namely the Wilcoxon two-sample test, to test the validity of research hypothesis H1 [49]. This test is one of the most widely used nonparametric methods in mathematical statistics. It is used as a non-parametric alternative to the parametric *t*-test for two independent sample sets, i.e. the hypothesis being tested is the following null hypothesis.

$H_{0}$: *Both sample sets come from the same base set, i.e. there is a difference between the two samples (males and females) in responses to the question “Satisfaction with the municipality/city you live in. Please indicate on a scale of 0-10” there is no statistically significant difference.*

We will test the null hypothesis against the alternative hypothesis:

$H_{1}$: *The sample sets do not come from the same base set, i.e. there is a statistically significant difference between the two samples (males and females) in responses to the question “Satisfaction with the municipality/town you live in”.* We use the following statistic (equation (1)) as a test criterion

(1) $$Z=\frac{U_{1}-\frac{1}{2}m\cdot n}{\sqrt{\frac{m\cdot n}{12}\left(m+n+1\right)}}\text{,}$$

which has an asymptotically normal distribution N(0,1) under the validity of the null hypothesis $H_{0}$. We reject the null hypothesis $H_{0}:\mu_{1}=\mu_{2}$ at the significance level of α in favour of the two-sided alternative hypothesis $H_{1}:\mu_{1}\neq \mu_{2}$, if $\left|\;Z\right|>u_{\alpha }\text{,}$ where $u_{\alpha }$ is the critical value of the normal normalized distribution. In our research, Wilcoxon two-sample test was used to test for statistically significant differences between the male-female groups in each observed character. Wilcoxon two-sample test [50] was conducted using STATISTICA software. First, we will test the statistical significance of the differences between the two sets on the question of satisfaction with the place. After entering the input data in the computer we obtained the output report with the following results for the chosen Wilcoxon two-sample test: the value of the test criterion *Z* and the *p*-value, which is the probability of error made when we reject the hypothesis being tested.

**Fig. 8:**
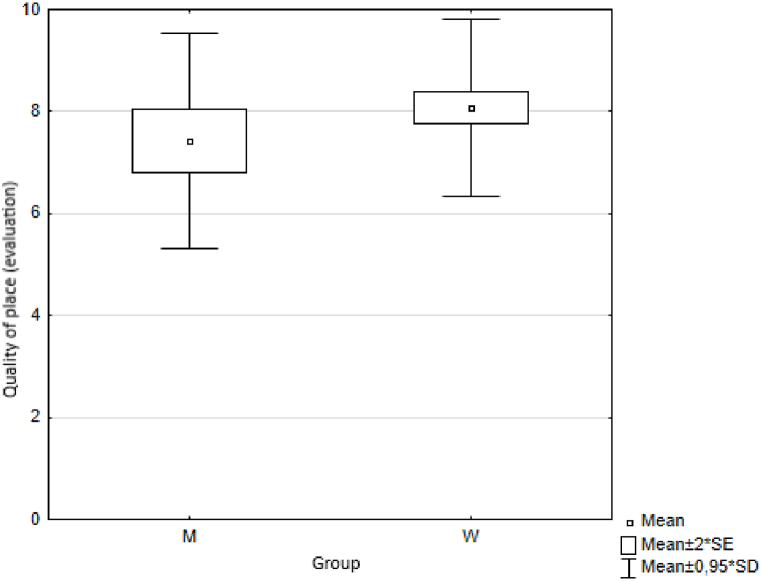
Average values of answers to the question on the quality of the place.

**Fig. 9:**
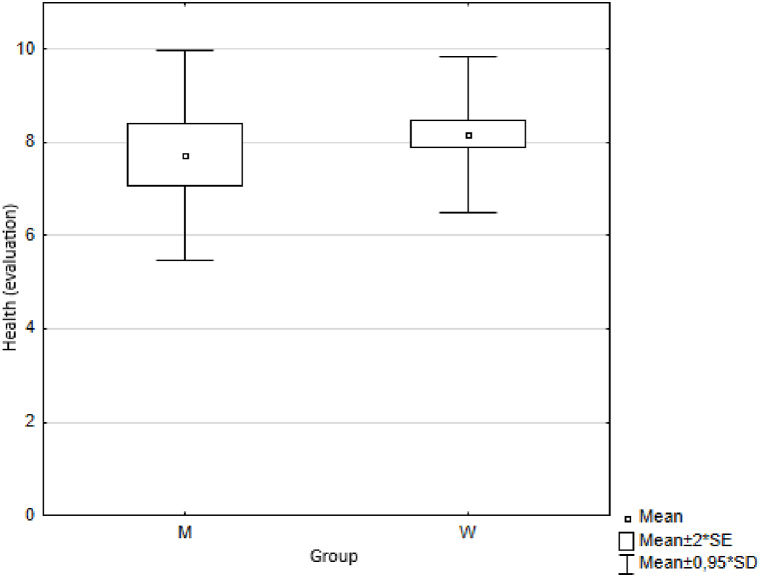
Average values of answers to the question on health.

**Fig. 10:**
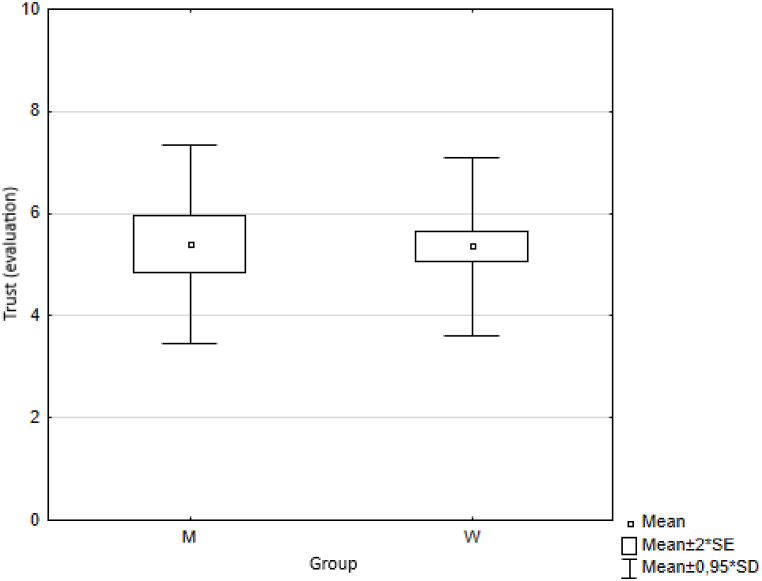
Average values of answers to the question on trust.

**Fig. 11:**
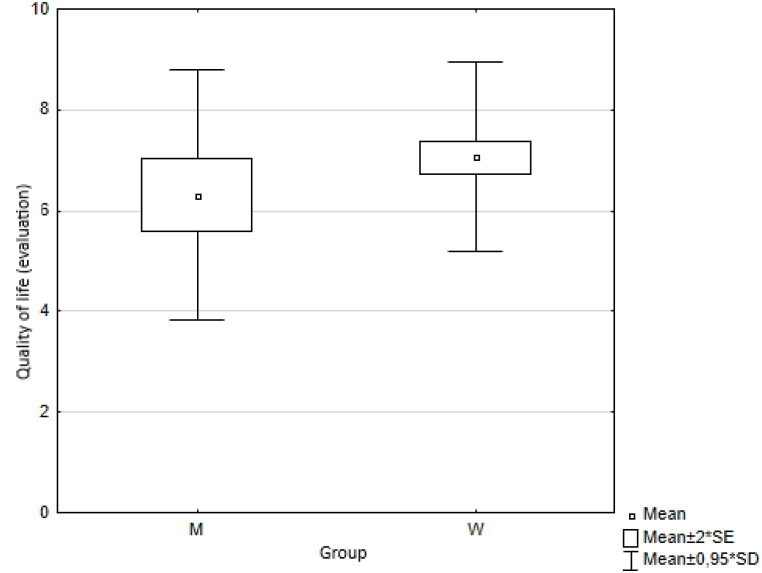
Average values of answers to the question on the quality of life.

**Fig. 12:**
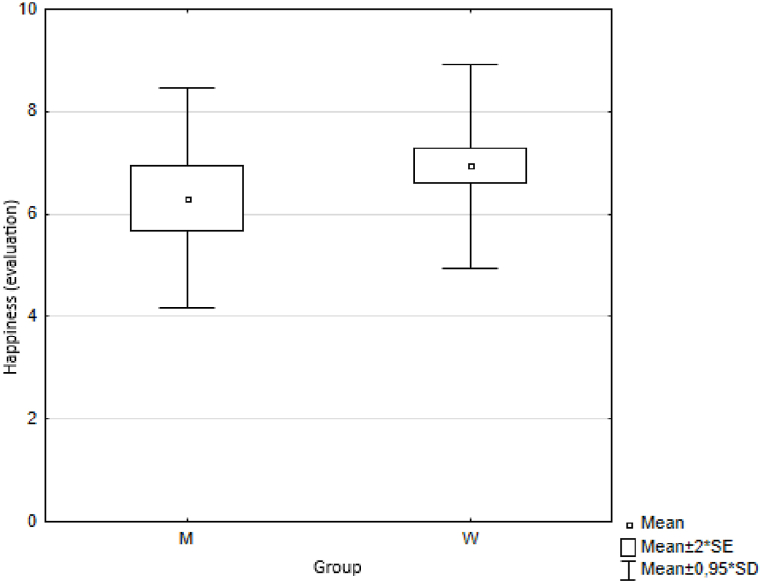
Average values of answers to the question about happiness.

**Fig. 13:**
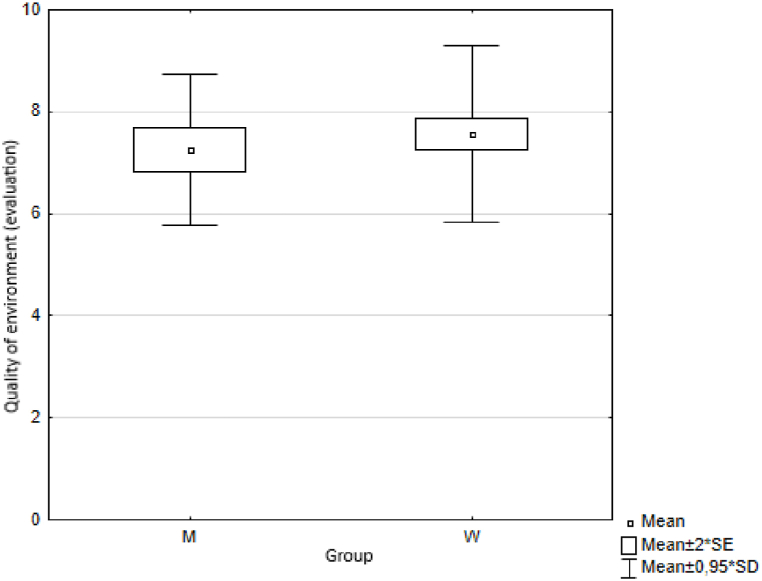
Average values of answers to the question of environment quality.

Using the Wilcoxon two-sample test we first analysed the results obtained by two groups of respondents (men and women) to the question: "*How satisfied are you with the municipality/city where you live. Please indicate on a scale of 0 - 10”*. Using the Wilcoxon two-sample test the value of the test statistic was Z = −1.972 and the probability value *p* = 0.049. Based on the calculated value of probability p = 0.05, we reject the hypothesis H0 at the level of significance. We accept the alternative hypothesis H1, i.e. the observed differences between men and women in the answers to the question "*Satisfaction with the municipality/city where you live. Please indicate on a scale of 0 - 10*″ are statistically significant. Specifically to the question *“Satisfaction with the municipality/city you live in. Please indicate on a scale of 0 - 10”*, men and women answered statistically significantly differently (Fig. 8). We followed a similar procedure to test the statistical significance of differences between men and women in their responses to other questions on trust, health, quality of life, happiness and environmental quality (Fig. 9, Fig. 10, Fig. 11, Fig. 12, Fig. 13).

The results are provided in Table 5.

**Table 5:** Wilcoxon two-sample test results.

Variables	Z	*p*
trust	0.496	0.620
health	−0.584	0.559
quality of life	−1.597	0.110
happiness	−0.584	0.559
quality of place	−1.703	0.089
quality of environment	−1.757	0.079

In the following, we were interested in the correlation (degree of dependence) between the observed variables - responses to the questions, both for men and women. Given that the above selections do not meet the assumption of a normal distribution of the observed variables, we used the coefficient of rank correlation to determine the degree of dependence between the above variables. Assume that we observe two ordinal variables *X* and *Y* on the elements of the set with range *n*. Let the character *X* assign to the elements of the set the order $x_{1},x_{2},\cdots ,x_{n}$ and the character *Y* the order $y_{1},y_{2},\cdots ,y_{n}$. The degree of dependence between variables *X* and *Y* is expressed by Spearman's rank correlation coefficient. It is defined by equation (2):

(2) $$R=1-\;\frac{6\sum_{i=1}^{n}d_{i}^{2}}{n\left(n^{2}-1\right)},\text{where}\;d_{i}=x_{i}-\;y_{i},\text{pre}\;i=1,2,\ldots ,n\text{.}$$

The coefficient of the rank correlation *R* takes values from the interval ⟨−1,1⟩ and the interpretation of its values is the same as for the Pearson correlation coefficient. We calculated the values of the Spearman's rank correlation coefficient between the values of the responses to each question for both males and females. The STATISTICA program was used for the calculation. The results are presented in Table 6, Table 7 and Table 8.

**Table 6:** Spearman's rank correlation coefficient values (male).

Variables	Trust	Health	Quality of life	Happiness	Quality of place	Quality of environment
trust	1.000					
health	0.243	1.000				
quality of life	0.311*	0.388*	1.000			
happiness	0.305*	0.418*	0.821*	1.000		
quality of place	0.186	0.235	0.202	0.196	1.000	
quality of environment	0.367*	0.217	0.495*	0.489*	0.428*	1.000

Note: * statistically significant value.

**Table 7:** Verbal assessment of predictors.

Correlation value	Verbal Indication of Correlation	Verbal Indication of the Predictor
≤0,09	No correlation	None
0,10 - 0,19	Very small correlation	None
0.20–0.29	Small correlation	None
0.30–0.49	Medium correlation	Predictor
0.50–0.69	Large correlation	Strong predictor
0.70–0.89	Very large correlation	Very strong predictor
0.90 ≥	Near perfect correlation	Near perfect predictor

**Table 8:** Spearman's rank correlation coefficient values (female).

Variables	Trust	Health	Quality of life	Happiness	Quality of place	Quality of environment
trust	1.000					
health	−.019	1.000				
quality of life	0.175	0.455*	1.000			
happiness	0.172	0.484*	0.678*	1.000		
quality of place	0.045	0.254	0.364*	0.291	1.000	
quality of environment	0.153	0.280	0.282	0.336*	0.363*	1.000

Note: * statistically significant value.

In terms of rank correlation for men, quality of life reached statistical significance and thus correlated with four variables - trust, health, happiness and environmental quality. This was not reached for quality of place. To assess whether any of these variables is a predictor of quality of life or not, we have arbitrarily set a correlation value of 0.30 and higher according to the verbal assessment of the predictors [47] (Table 7).

According to this criterion, the predictors of men's quality of life are trust, health, happiness and the quality of the environment.

In terms of rank correlation for women, quality of life reached statistical significance and thus correlation with three variables - health, happiness and quality of place, but did not reach it for trust and environmental quality. Predictors of women's quality of life are health, happiness and quality of place.

In Table 9 we compared the measured values of quality of life and other variables before the pandemic, in the first and third wave, using only the variables that were measured in all tests.

**Table 9:** Values of quality of life and other factors before the pandemic, in the first and third wave of the pandemic.

Period	Trust	Health	Quality of life	Happiness	Quality of place	Safety
Pre-pandemic (2019)	6.1	8.6	7.4	7.0	7.0	8.0
The first wave of the pandemic (2020) averages	5.9	8.2	7.6	7.5	7.7	8.1
Third wave of the pandemic (2021)	5.4	8.0	6.8	6.8	7.9	8.1

Four findings result from a comparison of changes in values on a scale of 0–10 for quality of life and other variables before the pandemic, in the first and third wave:

(i) in the first wave of the pandemic, respondents declared higher values of quality of life and other variables than before the pandemic, with the exception of trust. A possible explanation is the enthusiasm and support of health professionals, which resulted in a positive lock-down of society.
(ii) the highest value in measurements was achieved by the variable satisfaction with health, which could be expected due to the fact that the quality of life was measured among university students, i.e. young, usually healthy people.
(iii) in terms of quality of life for the winner’ of the third wave of the pandemic in the Czech Republic the variable - place - can be denoted, whose value as the only variable in the third wave increased compared to the first wave and the period before the pandemic.
(iv) in terms of quality of life, the variable – trust - can be described as 'defeated' in the third wave of the pandemic in the Czech Republic. As the only measured variable, it reached a lower value in the first wave of the pandemic compared to the period before the pandemic and in the third wave compared to the first wave. At the same time, trust achieved the lowest measured values.

The position of the place in terms of living a good quality of life is also confirmed by the answers to the other two questions. In the first, we wanted respondents to mention one or two of the seven options that they thought had a negative impact on their quality of life. The options were following: (i) fear of contracting the COVID-19 disease, (ii) fear for the lives of loved ones, (iii) social isolation, (iv) emotional isolation, (v) online teaching, (vi) lack of money and (vii) dissatisfaction with the place where they live. In the second question, on the other hand, respondents were asked to indicate one or two of the seven options, which they considered had a positive impact on their quality of life. The options were following: (i) pandemic management, (ii) social support, (iii) emotional support, (iv) interest in studying, (v) willingness to be modest (vi) altruism and (vii) satisfaction with the place where they live. The negative and positive evaluation of the place is in Table 10. A positive evaluation of place for both men and women far outweighs the negative one.

**Table 10:** Effect of location as a variable with positive/negative impact on quality of life.

	Positive		Negative	
	abs. (from)	%	abs. (from)	%
Men	23 (53)	43.4	3 (53)	5.7
Women	76 (150)	50.7	11 (150)	7.3
Sum	99 (203)	48.8	14 (203)	6.9

## 4 Discussion

The paper had two objectives (i) to measure the quality of life and related variables in the third wave of the pandemic and to compare the values found in the third wave with those in the first wave of the pandemic, and (ii) to investigate which of the variables related to quality of life are predictors of the quality of life.

Measured values of quality of life and related variables are shown in Table 4. The highest value is reached by health, which is not surprising given the age of the respondents. Values are high in quality of place and quality of environment. The validity of the data obtained will be enhanced by the comparison with the period before the pandemic and with the first wave of the pandemic (Table 9). The quality of life in the first wave of the pandemic increased compared to the pre-pandemic period, which Murgaš and Petrovič [10] considered as a continuation of the improvement in the quality of life after 2016. We observe a similar trend for happiness. Trust declined over the observed period. The effect of trust on quality of life is considered robust [51,52], its lowest measured values among all variables for Czech university students contradict this assumption. The low values and their decline imply that trust is the loser of the third wave of the pandemic.

The opposite of low and declining trust is quality of place, expressing satisfaction with the city or municipality in which the respondent lives. Values which were already high before the pandemic increased in both the first and third waves of the pandemic. The above findings are confirmed by the responses to two supplementary questions summarized in the quality of life measure in the third wave of the pandemic (Table 10). Satisfaction with the city or municipality where respondents live outweighed dissatisfaction by a ratio of 48.8%: 6.9%. The high values of quality of place confirmed by the responses to two related questions on factors with negative/positive impact on quality of life mean that cities and municipalities, i.e. the places where respondents live, are the winners of the third wave of the pandemic.

This brings us to the term 'place', which represents the contribution of geography to the social sciences, similar to the contribution of social capital to sociology. Place itself is a neutral term, in connection with the sense of place or its absence, a positive evaluation of the place, i.e. 'topophilia', arises. In the absence of sense of place, on the other hand, a negative assessment of the place, i.e. 'topophobia' arises [53]. McCunn and Gifford [54,55] enrich the concept of sense of place with the concept of 'imageability'. Place, sense of place and imageability are terms of environmental psychology, they refer to the objective dimension of quality of life. This is 'geography matter'.

Psychologist Peter J. Rentfrow combined psychology with geography into the concept of 'Geographical Psychology' by taking the adjective 'geographical' into psychology [24,56,57]. Johnston (online) [58] points out: “*The modern academic* discipline *of geography is rooted in ancient practice, concerned with the characteristics of places, in particular their natural environments and peoples, as well as the relations between the two”.* There are many definitions of geography; they have in common their understanding of man's relationship with the environment in which he lives. Florida and Melander [59] use the term Psychogeography [of creativity], in the paper we use the term geographical psychology [60].

The raison d’être of geographical psychology is researching the geographical organisation of psychological phenomena. Individual parts of psychology - personality, social, evolutionary, cultural, environmental, and comparative - and their development are significantly influenced by social, economic, political and ecological factors, which are spatially differentiated [24]. These factors, together with the physical environment, create a geographical environment, which manifests itself at different levels.

We believe that quality of life research is a topic par excellence for geographical psychologists. Holistic understanding of quality of life [[61], [62], [63]] and geographical psychology of quality of life can be considered as two sides of the same coin.

The second objective was to find out which of the factors related to quality of life are significantly correlated with it and are therefore predictors of it. We start with the verbal expression of numerical values of correlations [64]. As a criterion of significance, we set a mean and a higher value of the coefficient of the rank correlation (0.30–0.49); a factor with this correlation is a predictor of quality of life. A correlation value of 0.50–0.69 (large) means that the factor is a strong predictor of quality of life. Achieving a correlation value of 0.70–0.89 (very large) means that the factor is a robust predictor of quality of life.

According to this distribution, for men, predictors of quality of life include trust, health, and environmental quality. Happiness is a robust predictor. For women, health and quality of place are predictors of quality of life, and happiness is a strong predictor. The low values of the coefficients of the rank correlation of trust confirm that trust is the loser of the third wave of the pandemic among Czech female university students. On the other hand, the correlation of quality of place and quality of life despite expectation is small (0.202) for males and medium (0.364) for females.

### 4.1 Implications

#### 4.1.1 Implications for the epistemology of quality of life

From an epistemological point of view, a key finding of our paper is the knowledge of the high measured value of the place variable. One of the few generally accepted findings in the epistemology of quality of life is that it consists of two dimensions - subjective and objective. As the quality of life is rated as above average in most countries (on a Cantril scale of 0–10, a value of 6 or more), the subjective dimension tends to be identified with well-being. According to Murgaš [20] (2016: 311), “*well-being expresses the subjective, emotional survival and evaluation of satisfaction with its own life*”. The subjective dimension expresses the quality of a place, i.e. the extent to which a place meets the preconditions for experiencing a good life. The objective dimension is the more important of the two dimensions [[65], [66], [67]], but both dimensions are essential.

The second generally accepted finding is that the concept of quality of life is intertwined with the concept of 'good life'. When we evaluate the quality of our life we evaluate how good it is. The insight from our paper about the importance of quality of place in times of pandemic allows us to state that “quality of life means a *good life* lived in a *good place*”. The high measured values of satisfaction with the place confirm this.

#### 4.1.2 Implications for public policy

Quality of life has become a part of public life, and politicians at all hierarchical levels have included a commitment to improve it to their agendas. The measure of the success of public policy is the measurement of quality of life, or the related concepts of well-being or happiness. At the country level, rankings are issued by various bodies. In Table 1, we provided an overview of the quality of life or happiness measurements in the countries with the highest scores, issued by the media [31,32], a global database [33], a think-tank [34,37], and a UN component [35,40]. For comparison, we also present the ranking of Czechia.

Table 1 clearly shows that the ranking of the top performing countries is very stable, with Finland, Denmark, Norway, Switzerland, the Netherlands and Germany being included in all five measurements, Iceland in four of them. Czechia performs very well in these measurements, confirming its position as a leader among the post-transition countries of Central and Eastern Europe. From a regional point of view, the position of the Scandinavian countries is remarkable, the other remarkable thing is that the only country with a high population but also economic strength is Germany. The results of smaller countries should be inspiring and motivating for other post-transit countries with smaller populations.

In addition to measuring the quality of life in countries, attention is also paid to measuring the quality of life in the world's major cities. Petrovič and Murgaš [68] report the ranking of cities in the Global Liveability Index from 2015 to 2021. Until the start of the pandemic in 2019, the ranking of cities was characterized by a similar stability as the ranking of countries, but for 2021 there were significant changes in the ranking.

The above stated examples of quality of life measurement and stability of rankings are inspiring for public policy. It turns out that good public policy on a country level (Table 1) brings very good long-term results.

#### 4.1.3 Implications for geography

Quality of place and quality of environment can be considered as close but not interchangeable in terms of quality of life. The recognition that quality of place and quality of environment are the 'winners' of the third wave of the pandemic in terms of quality of life of Czech university students has an impact on geographical knowledge as well as strengthening the relevance of this science. Geography is the only science that deals with all qualitative phenomena [69], which is both its weakness and its strength.

Geography is concerned with the Earth's landscape, comprising five groups of overlapping geospheres. Three geospheres are studied by the life and non-living sciences, the fourth geosphere by the social, human and technical sciences. The fifth, immaterial geosphere, made up of the noosphere and cybersphere, is studied by philosophy, humanities and cognitive sciences [70]. Quality of life refers to all geospheres, however, according to a well-known definition [Quality of life is] “*a person's cognitive and affective evaluations of his or her life*” [71], the fifth sphere plays the most significant role in it.

The element in which the concepts of quality of life and geography intersect is place. Geographers consider place to be one of the five key themes of geography [72]. When geographers state that quality of life is spatially differentiated, they are expressing its value in particular places. People live their everyday lives in the space constituted by places [53] whatever their size. There is considerable evidence that 'place matters' [73]. This implies a distinction between place and space; place is part of space. If a place is a neighbourhood of a city, the space is the city. If the place is a city, the space is a district, a region or an entire state. The dichotomy of place and space as key elements of the human world was already described by Aristotle [70]. An inherent characteristic of the quality of life is its dichotomousness. Quality of life is both a goal and a path to the fulfilment of this goal. It is both subjective and objective.

Oťahel et al. [74] consider it essential for human geographers to engage in the study of the ways in which the material environment shapes social relations. The geographical study of quality of life is also dichotomous. On the one hand, geographers explore how quality of life is spatially differentiated; on the other hand, they investigate how the environment influences quality of life. The complexity of environmental influence at the level of Czech districts was pointed out by Murgaš and Klobučník [75].

There are two limitations in the paper. The first limitation is the fact that the target group on which the research was focused was represented by university students, who are a specific demographic group. Future research should focus on all demographic groups. The second limitation is the lack of coverage of the entire territory of Slovakia, which can only be solved by collecting data using the face-to-face method.

## 5 Conclusion

In hypothesis H1, we assumed differences in quality of life values between male and female students during the third wave of the pandemic. The hypothesis was confirmed, the mean quality of life values of female students (7.13) are higher than those of male students (6.43). The paper had two objectives. In the first we measured the quality of life and related factors in the third wave of pandemic on a scale of 0–10. We identified trust, health, happiness, quality of place and quality of environment as related factors. Health, quality of place, and quality of environment scored the highest. The lowest value was measured for trust. Therefore we consider trust to be the loser of the third wave of the pandemic. We compared the measured values in the third wave with those in the first wave of the pandemic. Values for quality of life and related factors eroded to lower values in the third wave, except for quality of place, the value of which improved. Therefore, we consider quality of place as the winner of the third wave of the pandemic.

The second objective was to find out which of the factors related to quality of life are significantly correlated with it and are therefore predictors of it. The criterion of significance was the mean and higher value of the rank correlation coefficient according to de Vaus [64]. For both men and women, health is a predictor of quality of life, and happiness is a robust predictor. Quality of place is a predictor of quality of life only for women.

## Ethical approval

Ethical approval was obtained from the Ethic Committee of the Constantine the Philosopher University in Nitra (chairman Prof. dr. M. Bauerová). The Ethics Committee stated and confirmed that the research does not contradict any ethical rules and confirms that all respondents were informed about the use of their answers. The questionnaire was filled out anonymously. All respondents were initially informed about the objectives of the research and the use of the questionnaire. By filling out the questionnaire, they agreed to its evaluation.

## Data availability statement

Data included in article/supp. material/referenced in article.

## Research funding

This work was supported by the Ministry of Education of the Slovak Republic and the Slovak Academy of Science [grant number 10.13039/501100006109VEGA 1/0578/24].

## CRediT authorship contribution statement

**František Murgaš:** Writing – original draft, Supervision, Methodology, Formal analysis, Conceptualization. **František Petrovič:** Writing – review & editing, Writing – original draft, Methodology, Funding acquisition, Conceptualization. **Anna Tirpáková:** Writing – original draft, Validation, Software, Formal analysis, Data curation.

## Declaration of competing interest

The authors declare that they have no known competing financial interests or personal relationships that could have appeared to influence the work reported in this paper.

## Footnotes

1 The *p*-value is the probability of the error we make when we reject the hypothesis $H_{0}$ being tested in favour of the alternative hypothesis $H_{1}$. If this probability is less than 0.05 or 0.01, we reject the tested hypothesis $H_{0}$ at the significance level of α=0,05 or α=0,01. Otherwise, we do not reject the hypothesis tested $H_{0}$.
