# Supplementary material for: Quality of place as the winner of the third wave of the COVID-19 pandemic in terms of quality of life. Will this knowledge strengthen the development of geographical psychology?
Source: Heliyon. 2024 Feb 10;10(4):e26261. doi: 10.1016/j.heliyon.2024.e26261 (PMC10881370; doi:10.1016/j.heliyon.2024.e26261)
Supplement: Multimedia component 1 [file mmc1.docx]

**Questionnaire**

- Trust. We measured trust by asking "How much do you trust people? Please indicate on a scale of 0-10, where 0 means you don't trust anyone and 10 means you trust everyone".

- Health. We measured health by asking "Are you healthy? Please indicate on a scale of 0-10, where 0 means you have a life-threatening illness or have suffered a life-threatening accident and 10 means you are absolutely healthy".

- Quality of life. We measured quality of life by asking „What is your quality of life? Please indicate on a scale of 0-10, where 0 is the worst possible and 10 the best possible quality of your life".

- Happiness. We measured happiness by asking "Are you happy? Please indicate on a scale of 0-10, where 0 means you are completely unhappy and 10 means you are completely happy".

- Quality of place. We measured quality of place by asking: "What is the quality of the place where you permanently live in terms of the conditions for living a good life? Please indicate on a scale of 0-10, where 0 means that the quality of the place is the worst possible, I want to move away and 10 means that the quality of the place is the best possible".

- Quality of environment. We measured quality of environment by asking: "What is the quality of the environment in the place where you live permanently? Please indicate on a scale of 0-10, where 0 means that the quality of the environment is the worst possible and 10 means that the quality of the environment is the best possible".
